# Effects of probiotics, prebiotics, and synbiotics on the growth performance, biochemical indexes, and gut morphometry of turkeys

**DOI:** 10.3389/fphys.2025.1703083

**Published:** 2025-11-19

**Authors:** A. S. Sulaiman, B. A. Hussaini, V. A. Uyanga

**Affiliations:** 1 Department of Biotechnology, Faculty of Agriculture and Veterinary Science, Mewar University Gangrar, Chittorgar, India; 2 Department of Veterinary Public Health and Preventive Medicine, Faculty of Veterinary Medicine, Bayero University Kano, Kano, Nigeria; 3 Department of Agriculture and Environmental Studies, Lincoln University of Missouri, Jefferson, MO, United States

**Keywords:** prebiotics, probiotics, synbiotics, turkey poults, growth performance, nutrientdigestibility, immunity

## Abstract

This study investigated the effects of dietary supplementation with probiotics, prebiotics, or synbiotics on the growth performance, immune function, and gut health in turkeys. A total of 180 turkeys were allocated into four dietary groups: basal diet (control group); basal diet supplemented with 1.0 × 108 CFU/g *Lactobacillus* acidophilus and *Bacillus subtilis* 1.0 × 108 CFU/g (Probiotic group); basal diet supplemented with 1% inulin and 0.5% Mannan oligosaccharides (Prebiotic group) or basal diet supplemented with both prebiotic and probiotic mixtures (Synbiotic group), and fed for 12 weeks. Growth performance was evaluated at 4–10 weeks (phase 1) and 10–16 weeks (phase 2), then blood and tissue samples were collected at the end of the study to assess immunological, biochemical parameters, and intestinal morphometry. Compared to the control group, the body weight gain of the probiotics and synbiotic groups were significantly higher at phase 2 (P < 0.05), and this persisted with synbiotic supplementation during the overall phase (P = 0.05). Similarly, the feed intake of turkeys during the overall phase was improved in the probiotic, prebiotics and synbiotics groups compared to the control group (P < 0.05). Carcass yield remained unaffected, but the spleen and bursa weights were significantly higher in the probiotics and synbiotic groups (P < 0.05). Blood analysis revealed elevated white blood cell counts and total cholesterol in turkeys fed probiotic, prebiotics and synbiotics relative to the control group (P < 0.05). Duodenal morphology showed no significant differences among treatment groups, whereas, the *Lactobacillus* counts were significantly higher in probiotics and synbiotics-fed turkeys compared to the control group (P < 0.05). Crude protein digestibility was higher with prebiotics and synbiotics supplementation, and the probiotic diet further increased nitrogen retention in turkeys (P < 0.05), compared to the control group. Additionally, behavioral assessments indicated increased activity of synbiotics-fed turkeys compared to the control group (P < 0.05). Overall, synbiotics emerged as an effective dietary intervention, providing the synergistic actions of both pre- and probiotics on the growth performance, immune functioning, and nutrient utilization, thus, underscoring their potential as a strategic nutritional supplement for turkey production.

## 1 Introduction

Global poultry meat output reached 146 million tonnes in 2023, increasing by ∼1.9% yearly. It is projected that global poultry production will grow by 16%, and account for about 43% of all meat protein consumed by 2033 (OECD and Food and Agriculture Organization of the United Nations, 2024). With the increase in demand and supply for poultry meat, turkey production has witnessed positive growth prospects, with the global turkey industry striving to produce high-quality meat at low costs with emphasis on increasing production efficiency, sustainability, and animal welfare (Cloft et al., 2018). Improvements in genetic selection practices, husbandry practices and disease management accrue significant progress within the turkey industry resulting to improved growth rate, higher feed conversion rate and higher meat output (Shehata et al., 2024). However, the turkey industry still faces various challenges, including high mortality during rearing (Institute of Poultry Diseases et al., 2021; Gernat et al., 2021), increase in feed cost, disease outbreaks, limited veterinary products, animal welfare and health concerns, intense global competition, food safety concerns, environmental issues, and regulatory changes, which affects economic outcomes (Dittoe et al., 2019). The ban on in-feed antibiotics has also further raised concerns in turkey production, given that only a limited number of veterinary products are approved for treating turkeys as food-producing animals (Gulmez et al., 2019).

With the ban on antibiotic growth promoters, interest in alternative solutions has increased (Feye et al., 2016). Probiotics, which are live microorganisms that promote gut health, have emerged as popular substitutes (Gulmez et al., 2019; Khomayezi and Adewole, 2022). They improve the microbial balance, enhance immune responses, and increase resistance against pathogens (Gulmez et al., 2019). Probiotics, including strains of *Lactobacillus*, *Bacillus*, and *Enterococcus*, have been shown to directly modulate the gut microbiota by improving nutrient absorption, strengthening the intestinal barrier through increased villus height and tight junction proteins, and enhancing immune responses by reducing pro-inflammatory cytokines (Cheng and Ning, 2023; Idowu et al., 2025). Similarly, prebiotics, which are non-digestible oligosaccharides are known to stimulate the growth of beneficial gut bacteria and promote overall gut health (Lipiński et al., 2021). For instance, mannan-oligosaccharides have been demonstrated to reduce *Salmonella* colonization by acting as a decoy receptor to promote microbial production of SCFAs, while inhibiting pathogenic bacteria (Sharma et al., 2018). When digested in the colon, prebiotics and probiotics produce short-chain fatty acids, which lower pH levels, creating an unfavourable environment for harmful bacteria while improving mineral absorption (Lipiński et al., 2021; Markowiak and Śliżewska, 2017).

When used together as synbiotics, probiotics and prebiotics provide additional benefits by improving nutrient digestion and enhancing gut health, leading to improved performance in poultry (Mohammadigheisar et al., 2019; Vahabi-Asil et al., 2017). Studies demonstrate that synbiotics significantly improve productivity metrics such as egg mass and shell thickness in layers, and it also enhances the feed efficiency in broilers by ensuring probiotic viability and activity, improving villus architecture, nutrient absorption, and immune function (Toghiani et al., 2024; Roberfroid, 2018). However, while probiotics and prebiotics have been extensively studied in broilers and laying hens, research on their use in turkeys is limited and has shown inconsistent findings (Gulmez et al., 2019).

Post-antibiotic ban, probiotics (e.g., *Lactobacillus spp*.) and prebiotics (e.g., inulin) have gained traction as alternatives (Feye et al., 2016), yet turkey-specific formulations remain underexplored. Only 8/63 poultry probiotic trials (2020–2023) focused on turkeys with 75% using broiler-derived strains a potential mismatch for turkey gut ecology, with only 12% of probiotic/prebiotic trials focusing on *Meleagris gallopavo* (Gulmez et al., 2019; Khomayezi and Adewole, 2022). Utilizing feed additives like probiotics, prebiotics, and synbiotcs may help improve the performance of poultry birds by modifying the intestinal microbiota, boosting immune response, and safeguarding intestinal integrity (Dittoe et al., 2019). The present study investigated the effects of supplementing probiotics, prebiotics, or synbiotic on the growth performance, carcass traits, immune responses and gut morphology of turkey poults. It was hypothesized that synbiotic supplementation would be more efficacious in improving the growth performance, immune response, and gut health of turkeys than providing probiotics or prebiotics individually. This is based on the premise that the prebiotic component selectively stimulates the growth and activity of the supplemented probiotic bacteria, leading to a more profound and stable modulation of the gut microbiome, which in turn enhances nutrient absorption and immune function.

## 2 Materials and methods

The research was conducted at the research farm of the Department of Agriculture in collaboration with the Department of Life Sciences at Mewar University, India. Ethical approval was obtained from the Local Ethics Commission for Experiments with Animals at Mewar University, India.

### 2.1 Experimental design

A total of 180 male broad-breasted bronze poults, at 30 days old, were obtained from Tamil Nadu Agricultural University (TNAU), India. The poults were weighed individually (average initial body weight: 0.9 ± 0.2 kg) and distributed at random among 12 wooden shaving-lined enclosures (250 × 250 cm). The experiment had 4 treatments, 3 replicates with 15 turkeys per replication. The birds were provided with 16 h of light and 8 h of darkness (16 L: 8D). The experimental groups were composed of turkey poults fed with basal diet (control group); basal diet supplemented with 1.0 × 10^8^ CFU/g *Lactobacillus acidophilus* and *Bacillus subtilis* 1.0 × 10^8^ CFU/g (Probiotic group); basal diet supplemented with 1% inulin and 0.5% mannan oligosaccharides mixture (Prebiotic group) or basal diet supplemented with both prebiotic and probiotic mixtures (Synbiotic group), and the study lasted for 12 weeks. Based on prior evidence that *Lactobacillus acidophilus* thrives on inulin/MOS substrates (Lambo et al., 2021), we selected this combination to maximize synergistic effects on gut health.

The experimental diets were prepared at 2 phases (week 4–10; and week 11–16) according to NRC recommendations to meet the nutritional requirements of turkey poults (Table 1) (National Research Council, 1994). Feed and water were provided *ad libitum* during the study. To preserve the stability of the feed additives, the prepared feed was sealed in airtight bags and kept in a cool, dry conditions at a temperature of 4 °C.

**TABLE 1:** Nutrient composition of experimental diets for turkeys.

Ingredients	Control	Probiotics	Prebiotics	Synbiotics
Phase 1 (4–10 Weeks)				
Maize (%)	60	60	60	59.7
Sunflower meal (%)	30	30	30	29.7
Wheat bran (%)	5.95	3.95	4.45	4.45
Probiotics	None	1.0 × 10^8^ + 1.0 × 10^8^ CFU/g	None	0.5 × 10^8^ + 0.5 × 10^8^ CFU/g
Prebiotics	None	None	1% + 0.5%	0.5% + 0.25%
Limestone (%)	1.2	1.2	1.2	1.2
Dicalcium phosphate (%)	1.8	1.8	1.8	1.8
Vitamin premix	0.2	0.2	0.2	0.2
Mineral premix	0.1	0.1	0.1	0.1
Salt (%)	0.3	0.3	0.3	0.3
Methionine (%)	0.3	0.3	0.3	0.3
Lysine (%)	0.15	0.15	0.15	0.15
Phase 2 (10–16 Weeks)				
Maize (%)	55	55	55	54.7
Sunflower meal (%)	25	25	25	24.7
Wheat bran (%)	16.38	14.38	14.88	14.88
Probiotics	None	1.0 × 10^8^ + 1.0 × 10^8^ CFU/g	None	0.5 × 10^8^ + 0.5 × 10^8^ CFU/g
Prebiotics	None	None	1% + 0.5%	0.5% + 0.25%
Limestone (%)	1.0	1.0	1.0	1.0
Dicalcium phosphate (%)	1.6	1.6	1.6	1.6
Vitamin premix	0.2	0.2	0.2	0.2
Mineral premix	0.1	0.1	0.1	0.1
Salt (%)	0.3	0.3	0.3	0.3
Methionine (%)	0.3	0.3	0.3	0.3
Lysine (%)	0.12	0.12	0.12	0.12
Total (%)	100	100	100	100

### 2.2 Growth performance

The initial body weight (IBW), final body weight (FBW), body weight gain (BWG), feed intake (FI), and feed conversion ratio (FCR) were determined. The IBW was recorded at the start of the study, while FBW was assessed on days 42 and 84 of the trial. To calculate body weight gain (BWG), the difference between FBW and IBW was used. The amount of feed left over from the amount offered was deducted over a certain period and used to calculate the average feed intake (FI). As a gauge of feed efficiency, the feed conversion ratio (FCR) was computed as the ratio of FI to BWG.

### 2.3 Blood collection

At the end of the trial, three (Shehata et al., 2024) birds per replicate of each treatment (n = 36) with representative body weights (±5% of group mean) were selected for blood collection. Blood samples were withdrawn from the wing vein of the birds into collection tubes containing 10 mg of ethylene diamine tetra acetic acid (EDTA), as an anticoagulant. Blood samples were aliquoted for haematological analysis and a portion was centrifuged for 20 min at 3,000 rpm for plasma collection. The resulting plasma was transferred into 2 mL Eppendorf tubes and stored at −20 °C until analysis.

### 2.4 Hematology and biochemical analyses

Blood metabolites, including total cholesterol, high-density lipoprotein (HDL), and low-density lipoprotein (LDL) levels, were measured in the plasma using a clinical chemistry analyser (Roche Cobas c111, Roche Diagnostics, Mannheim, Germany) (Roche Diagnostics, 2006). Blood components, including the red blood cells, white blood cells, and platelet counts were assessed using a haemocytometer (Neubauer Hemocytometer, Louis-Charles Malassez design). Under a cover slip, the diluted sample was fed into the hemocytometer chambers, making sure that the grid was filled without overflow. The hemocytometer was put under the microscope and the cells were counted using the proper magnifications (40x for RBCs and platelets, 10x for WBCs) (Rodak et al., 2020).

### 2.5 Tissue collection and organ weights

At the end of the trial, three (Shehata et al., 2024) birds per replicate were selected for euthanasia by electrically stunning and exsanguination in accordance with the AVMA Guidelines for the Euthanasia of Animals (Underwood and Anthony, 2020). Carcass were weighed individually, then the muscle and immune organs such as the spleen, bursa, and thymus were isolated and weighed to determine the organ weights (Sharma et al., 2018). To calculate the carcass yield, the carcasses were weighed and computed as a percentage of the live weight.

### 2.6 Gut morphometry

For histomorphological analysis, a 3-cm segment of the duodenum was collected from a standardized location immediately posterior to the duodenal loop. The duodenum was selected as it is the primary site for nutrient absorption in poultry and is highly responsive to dietary modifications (Teirlynck et al., 2009). To preserve the integrity of the mucosal structure, the intestinal segment was gently flushed with ice-cold phosphate-buffered saline (PBS) to remove digesta without applying any mechanical pressure that could damage the villi (Awad and Awaad, 2017). The segment was then preserved in 10% buffered formalin and embedded in paraffin, then sectioned at 5 µm thickness using a microtome. Hematoxylin and eosin (H&E) staining were applied to the prepared tissue sections. The villus height and crypt depth were measured under a microscope at ×40 magnification, then analyzed using ImageJ analysis software (Schneider et al., 2012).

### 2.7 Microbial composition

For microbial counting, the duodenum was carefully dissected from the gastrointestinal tract to avoid contamination, and the digesta was gently squeezed out into sterile containers using sterile forceps, placed in a sterile container, and chilled to 4 °C. Colony-forming units (CFUs) were counted via culture techniques. *Escherichia coli* (Eosin methyl blue agar, incubated aerobically for 24 h) and *Lactobacillus* (Man Rogosa Sharpe agar, incubated anaerobically for 48 h) were used to count the bacteria. In separate tubes, 9 mL of pre-reduced salt medium was filled with 1 mL of the digesta. The suspension was made via a 10^−1^ dilution, and successive dilutions were performed. The media was then cultured via serial dilutions of 10^−3^ and 10^−5^. A 0.1 mL sample from the dilution was plated onto a suitable medium to count the bacteria. Using a colony counter, discrete colonies on plates were counted, and the quantity of bacteria per 1 g sample (log_10_ CFU/g) was estimated.

### 2.8 Meat drip loss

A sample from the cranial portion of the right *Pectoralis major* (breast) muscle was excised for pH measurement immediately after slaughter (initial pH) and after 15 h of storage at 4 °C (ultimate pH), following standard meat quality assessment protocols for poultry (Mazzoni et al., 2015). The samples were placed individually in labelled, perforated plastic bags to allow drainage of exudates without external contamination. The bags containing the muscle samples were stored in a refrigerator set at 4 °C for 24 h. After storage, the final weight of each sample was recorded. Percentage drip loss was calculated as initial weight minus final weight of muscle divided by the initial weight * 100.

### 2.9 Meat pH

The pH of the breast muscle was measured by directly inserting the pH meter electrode into the thickest part of the muscle tissue, and the pH was recorded after the reading was stable. Two measurements were taken for each sample, and the average value was used to represent the meat pH. Each time the electrode was used, it was properly cleaned to prevent contamination. The pH of each sample was measured 30 min after the animals were killed (for the initial pH) and 15 h later (for the final pH). The electrode was placed straight into the thickest area of the breast tissue, ensuring that it was deep enough. After the pH meter was stable, the pH was recorded.

### 2.10 Fecal composition analysis

At the end of the trial, fecal samples from each treatment group were gathered using a clean spatula each day during a 3-day period. After the faeces samples were homogenized in plastic bags, the moisture content was determined by placing the samples in an oven at 80 °C for 48 h. Samples of the diet and excreta were air-dried and finely ground. The levels of crude protein, crude fat, crude fibre, nitrogen retention, and phosphorus retention were determined using standard operating procedures as previously reported (AOAC International, 2025).

### 2.11 Behavioural assessment

Bird behavior was observed and recorded between 08:00 and 10:00 h using scan sampling at 10-min intervals over a 2-h period. Feeding behaviour, such as feeding frequency and duration, were carefully observed and recorded (Hikvision Digital Technology Co. and Ltd, 2023). The number of times the turkeys consumed the feed and the duration of each feeding session were noted. Other behaviors such as walking, pecking, and resting were recorded as general activity levels.

### 2.12 Statistical analysis

To evaluate the main effects of dietary treatment, data collected were analyzed using a one-way analysis of variance (ANOVA) in a completely randomized design (CRD), with the dietary treatment as the main effect using the general linear model (GLM) procedure on Minitab 21 software (Minitab, 2023). The treatment means were compared via Tukey’s honestly significant difference test, treating dietary group as a fixed effect and pen as a random effect, which was used to establish statistical significance at *P* < 0.05.

## 3 Results

### 3.1 Growth performance

The growth performance of turkeys was analysed at two periods, phase I (4–10 weeks) and phase II (10–16 weeks) (Table 2). In Phase I, there were no significant differences in IBW and FBW among the treatment groups (*P* > 0.05), showing a similar starting point and similar growth outcomes across treatment groups. However, BWG showed significant differences (*P* < 0.05), where the control group was found to have the highest gain (2.43 kg/bird), while the probiotics (1.96 kg/bird) and prebiotics (1.98 kg/bird) groups had significantly lower gains. The FI and FCR showed no statistical differences (*P* > 0.05).

**TABLE 2:** Growth performance of turkeys fed with dietary probiotics, prebiotics or synbiotics supplement.

Parameters	Treatment groups				SEM	*P* - value
	Control	Probiotics	Prebiotics	Synbiotics		
Phase I (4–10 weeks)						
IBW (kg/bird)	4.17	4.31	4.32	4.16	0.120	0.684
FBW (kg/bird)	6.60	6.26	6.30	6.23	0.149	0.344
BWG (kg/bird)	2.43^a^	1.96^b^	1.98^b^	2.08^ab^	0.092	0.024
FI (kg/bird)	4.29	4.24	4.07	4.31	0.147	0.655
FCR	1.77	2.16	2.06	2.09	0.087	0.058
Phase II (10–16 weeks)						
IBW (kg/bird)	6.60	6.27	6.30	6.23	0.149	0.344
FBW (kg/bird)	9.85	11.03	10.34	11.22	0.315	0.052
BWG (kg/bird)	3.25^b^	4.43^a^	4.04^ab^	4.65^a^	0.251	0.019
FI (kg/bird)	6.02^b^	6.87^ab^	7.55^a^	7.21^a^	0.203	0.004
FCR	1.85	1.59	1.87	1.55	0.120	0.206
Overall period (4–16 weeks)						
BWG (kg/bird)	5.68^b^	6.39^ab^	6.02^ab^	6.73^a^	0.228	0.053
FI (kg/bird)	10.32^b^	11.12^a^	11.62^a^	11.49^a^	0.147	0.001
FCR	1.81	1.87	1.96	1.82	0.063	0.377

Keys: ^ab^Means with different superscript along the row are significantly different (*P ≤* 0.05).

IBW, initial body weight; BWG, body weight gain; FI, feed intake; FCR, feed conversion ratio.

In Phase II, no significant differences were observed in IBW and FCR (*P* > 0.05), consistent with Phase I results. For FBW, the results approached significance (*P* = 0.05), with Synbiotics (11.22 kg/bird) and Probiotics (11.03 kg/bird) groups having higher weights compared to the Control (9.85 kg/bird) and Prebiotics (10.34 kg/bird). Significant differences in BWG (*P* < 0.05) were evident, with Synbiotics (4.65 kg/bird) and Probiotics (4.43 kg/bird) outperforming the Control group (3.25 kg/bird). The FI also differed significantly (*P* < 0.05), with Prebiotics (7.55 kg/bird) and Synbiotics (7.21 kg/bird) groups consuming more feed compared to the Control group (6.02 kg/bird).

The overall performance of turkeys (4–16 weeks) revealed that the synbiotics group had the highest BWG (6.73 kg/bird) compared to the control group at (5.68 kg/bird), while the probiotic and prebiotic fed turkeys were intermediate (*P* > 0.05). Feeding turkeys with probiotics, prebiotics, and synbiotics significantly increased the FI relative to the control fed turkeys (*P* > 0.05). However, the different dietary treatments did not significantly impact the overall FCR of turkeys (*P <* 0.05).

### 3.2 Carcass and meat quality

Carcass weight and carcass yield were not significantly (*P* > 0.05) different among the treatment groups, suggesting that dietary inclusion of probiotics, prebiotics, or synbiotics did not markedly affect carcass characteristics (Table 3). However, breast muscle pH showed a significant difference (*P* < 0.05), with prebiotics-fed turkeys (5.87) having a higher pH compared to the probiotics group (5.13). Drip loss did not differ significantly (*P* > 0.05), indicating comparable water retention in the meat. The spleen weight was significantly higher in turkeys fed probiotics, prebiotics, and synbiotics, while the bursa of Fabricus weight was increased with probiotics and synbiotic diets compared to the control group (*P* < 0.05). In addition, the mortality rates were not significantly different (*P* > 0.05), showing that dietary treatments did not adversely affect turkey survival.

**TABLE 3:** Carcass and meat quality of turkeys fed with dietary probiotics, prebiotics or synbiotics supplements.

Parameters	Treatment groups				SEM	*P* - value
	Control	Probiotics	Prebiotics	Synbiotics		
Carcass weight (kg)	7.79	8.03	7.91	8.08	0.178	0.673
Carcass yield (%)	74.99	75.02	75.09	75.08	0.055	0.573
Breast muscle pH	5.70^ab^	5.13^b^	5.87^a^	5.30^ab^	0.149	0.027
Drip loss (%)	1.90	2.06	1.73	1.83	0.095	0.173
Spleen (g)	11.67^b^	14.67^a^	15.00^a^	16.33^a^	0.500	0.001
Bursa of Fabricus (g)	1.03^b^	1.40^a^	1.33^ab^	1.53^a^	0.076	0.010
Mortality (%)	6.66	2.22	2.22	4.43	3.327	0.753

Keys: ^ab^Means with different superscript along the row are significantly different (*P* < 0.05).

### 3.3 Haematological and biochemical profile

Table 4 shows that the WBC counts were significantly higher (*P* < 0.05) in the probiotics, prebiotics, and synbiotics groups compared to the control group. The RBC counts and platelet levels did not differ significantly (*P* > 0.05), indicating that dietary treatments had no effect on these parameters. The LDL levels showed no significant differences (*P* > 0.05), although the synbiotics group had the highest concentrations (118.67 mg/dL). The HDL levels also showed no significant differences (*P* > 0.05) but were considerably higher in the synbiotics group (62.67 mg/dL) than the control group. The TC levels were also significantly higher (*P* < 0.05) in turkeys receiving probiotics, prebiotics and synbiotics compared to the control group, indicating the potential effect of these treatments on lipid metabolism.

**TABLE 4:** Hematological and biochemical profile of turkeys fed with dietary probiotics, prebiotics or synbiotics supplements.

Parameters	Treatment groups				SEM	*P* - value
	Control	Probiotics	Prebiotics	Synbiotics		
WBC (x 10^3^/μL)	19.00^b^	22.67^a^	25.33^a^	22.67^a^	0.645	0.001
RBC (x 10^6^/μL)	2.43	2.67	2.53	2.87	0.133	0.197
Platelets (x 10^3^/μL)	23.33	24.67	23.67	23.67	1.013	0.808
LDL (mg/dL)	108.33	115.33	114.00	118.67	2.309	0.070
HDL (mg/dL)	55.33^b^	59.33^ab^	60.67^ab^	62.67^a^	1.615	0.063
TC (mg/dL)	190.00^b^	198.33^a^	196.00^a^	196.00^a^	1.054	0.003

Keys: ^ab^Means with different superscript along the row are significantly different (*P* < 0.05).

WBC, white blood cells; RBC, red blood cells; LDL, low-density lipoprotein; HDL, high-density lipoprotein; TC, total cholesterol.

### 3.4 Gut morphology and microbiota composition

The villus height, crypt depth, and villus height-to-crypt depth ratio did not show significant (*P* > 0.05) differences among the treatment groups (Table 5). However, *Lactobacillus* spp. counts were significantly higher (*P* < 0.05) in turkeys fed probiotics (7.53 × 10^6^) compared to the control group, indicating a beneficial impact of probiotics on microbiota composition. In addition, there were no significant differences in the *E. coli* population (*P* > 0.05).

**TABLE 5:** Duodenal morphology and microbial composition of turkeys fed with dietary probiotics, prebiotics or synbiotics supplements.

Parameters	Treatment groups				SEM	P - value
	Control	Probiotics	Prebiotics	Synbiotics		
VH(µm)	973.33	1,050.00	1,096.67	1,086.67	29.43	0.065
CD (µm)	180.00	190.00	206.67	190.00	6.665	0.112
VH/CD ratio	5.42	5.53	5.33	5.72	0.212	0.619
*L. spp* (CFU/g)	6.83×(10^6^)^c^	7.53×(10^6^)^a^	7.0×(10^6^)^bc^	7.3 (×10^6^)^ab^	0.079	0.001
*E. coli* (CFU/g)	1.23 × 10^4^	1.27 × 10^4^	1.2 × 10^4^	1.23 × 10^4^	0.144	0.990

Keys: ^abc^Means with different superscript along the row are significantly different (P < 0.05).

VH, villus height; CD, crypt depth; VH/CD, villus height to crypt depth ratio; CFU, Colony-forming unit, *E. coli* = *Escherichia coli*, L. spp = *Lactobacillus* spp.

### 3.5 Fecal nutrient composition

Table 6 presents the analysed nutrient composition of fecal samples. Crude protein content was significantly higher with synbiotic and probiotics-fed turkeys (*P* < 0.05), relative to the control groups. Nitrogen retention also differed significantly (*P* < 0.05), with probiotics-fed turkeys showing the highest retention (71.27%) compared to the control and synbiotics-fed groups. Crude fiber, crude fat and phosphorus retention did not show any significant differences among the treatment groups (*P* > 0.05).

**TABLE 6:** Fecal nutrient composition of turkeys fed with dietary probiotics, prebiotics or synbiotics supplements.

Parameters	Treatment groups				SEM	*P* - value
	Control	Probiotics	Prebiotics	Synbiotics		
Crude protein (%)	73.00^b^	78.00^ab^	79.67^a^	80.33^a^	1.312	0.016
Crude fibre (%)	59.00	59.33	61.00	61.00	1.509	0.693
Crude fat (%)	72.67	76.67	75.33	74.33	1.269	0.231
Nitrogen retention (%)	69.33^b^	71.27^a^	71.00^ab^	69.33^b^	0.500	0.023
Phosphorus retention (%)	56.00	58.00	58.00	59.00	1.607	0.626

Keys: ^ab^Means with different superscript along the row are significantly different (*P* < 0.05).

### 3.6 Behavioural assessment

**FIGURE 1:**
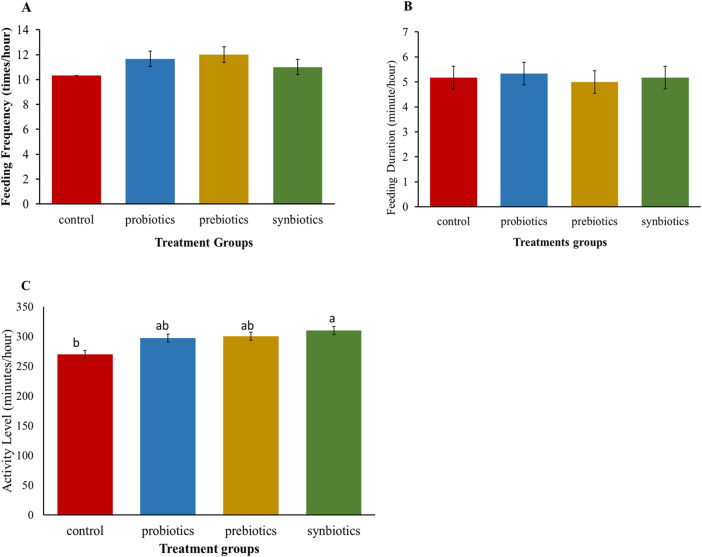
Behavioral responses of turkey birds fed with dietary probiotics, prebiotics or synbiotic supplements. **(A)** Feeding frequency, **(B)** Feeding duration, **(C)** General activity levels. Bars represent mean and SEM. ^*a, b,*^ Means with different superscripts on bar charts are significantly different (*P* < 0.05).

Feeding frequency (FF) and feeding duration (FD) did not show significant (*P* > 0.05) differences among the treatment groups (Figures 1A,B), suggesting that dietary treatments did not alter feeding behaviour of turkeys. However, other behavioural interactions showed considerable variation (Figure 1C) with turkeys in the synbiotics group being the most active compared to the control group.

## 4 Discussion

In the present study, the impacts of dietary supplementation with probiotic, prebiotic, and synbiotic were examined on the performance, gut morphology, nutritional digestibility, and behaviour of turkeys. Early development (4–10 weeks) and later growth (10–16 weeks) sages were the two phases used to assess turkeys’ growth performance. Body weight gains became evident in the later growing phase, with greater gains being the outcomes of dietary treatments that included probiotics and synbiotics, indicating improved food utilization and metabolic efficiency. This study validates the findings of Mookiah et al. (2014) and Lambo et al. (2021), who suggested that probiotics, prebiotics, and the combinations of probiotics with prebiotics increased the BWG, FCR, and overall productivity in relatively older chickens. Similarly, the overall feed intake increased with probiotics, prebiotics, and synbiotics supplementation, suggesting possible improvements in feed digestibility or appetite stimulation. The role of these supplements in enhancing the performance, nutritional absorption, and gut health has been extensively studied (Vahabi-Asil et al., 2017). Prebiotics operate as a substrate to increase probiotic activity, probiotics support beneficial bacteria to maintain microbial balance, and synbiotics combine the two strategies to produce advantages that work in concert (Patterson and Burkholder, 2003). In phase two and overall performance, the use of synbiotics had a synergistic impact that improved the growth performance beyond the effects of solo treatments (probiotics and prebiotics). This supports research showing that the simultaneous delivery of selective substrates and advantageous bacteria improves gut health, increases nutrient absorption, and reduces growth-limiting factors (Markowiak and Śliżewska, 2018). The findings corroborate the results of Abdelqader et al. (2020) and Li et al. (2016), who reported that *Lactobacillus acidophilus* and *Bacillus subtilis* increased the colonization of beneficial gut bacteria under heat stress, hence improving growth performance. Evidently, growth performance was positively impacted by both probiotic and prebiotic administration, but their combination produced the most consistent gains, especially at the later growth stages. These results align with previous studies suggesting that synbiotics and probiotics can positively influence gut health and nutrient absorption, leading to improved growth (Khomayezi and Adewole, 2022; Reuben et al., 2021). Overall, the findings underscore the potential of synbiotics and probiotics as effective dietary strategies for improving turkey growth performance.

Carcass analysis revealed no variations in drip loss, carcass weight, and carcass yield. Meat pH is a critical indicator of post-mortem glycolysis, influencing meat quality attributes such as water-holding capacity, tenderness, and color. In the present study, turkeys fed with prebiotic diets had considerably higher meat pH than probiotic fed turkeys. The observed differences in pH may reflect variations in post-mortem metabolic activity, potentially linked to the dietary modulation of gut microbiota and its impact on muscle metabolism (Gulmez et al., 2019; Elshaghabee et al., 2017). Furthermore, the observed increase in the relative weights of the spleen and bursa of Fabricius in the synbiotic group could indicate immune system stimulation. However, it is important to note that lymphoid organ hyperplasia is not exclusively a positive indicator; it can also be a response to subclinical infections, dietary antigens, or other physiological stressors (Fairbrother et al., 2005). Given the lack of clinical signs of disease and the improved performance in these birds, the observed changes may be linked to immune activation, but this interpretation is made with caution which is consistent with previous findings (Reuben et al., 2021; Kunvar et al., 2021). These may indicate an improved immune response since probiotics enhance the epithelial barrier and promote immunological function by inducing mucosal immunity (Bermudez-Brito et al., 2012). Through their interactions with macrophages, lymphocytes, epithelial cells, and dendritic cells, they alter the immune system and cause an increase in immunological responses (Ashraf and Shah, 2014).

Turkey WBC counts increased with the addition of probiotics, prebiotics and synbiotics, validating that the addition of probiotics and prebiotics enhances immunological responses (Asgari et al., 2016). This aligns with previous studies demonstrating that prebiotics, such as inulin and oligosaccharides, can enhance immune function by promoting the growth of beneficial gut microbiota, which in turn stimulates systemic immune responses (Schley and Field, 2002; Lomax and Calder, 2008). The increase in WBC counts may reflect enhanced immune surveillance and response, which is vital for maintaining health and preventing infections. However, it is important to note that excessively high WBC counts could also indicate inflammation or stress responses, which should be further investigated in future studies (Carr and Maggini, 2017). The increase in total cholesterol with the addition of probiotics, prebiotics and synbiotics may be attributed to the metabolic effects of gut microbiota modulation. Probiotics and prebiotics are known to influence lipid metabolism by altering bile acid reabsorption, cholesterol assimilation, and SCFA production (Ooi and Liong, 2010; Joyce et al., 2014). Synbiotics, which contain the combination of probiotics and prebiotics, have been shown to exert synergistic effects on lipid profiles. While the increase in total cholesterol in the treatment groups was statistically significant, the concurrent increase in HDL levels in these groups suggests a potential improvement in the overall lipid profile, which may mitigate cardiovascular disease risk (Grundy et al., 2019).

The use of probiotics and prebiotics enhances immune function and general health, possibly through improving nutrient absorption and altering the gut flora (Zheng et al., 2016). Evaluation of duodenal morphology revealed numerical trends indicating possible beneficial effects of probiotics, prebiotics and synbiotics in improving the villus height of turkeys better than the un-supplemented turkeys. Although insignificant, these findings are consistent with earlier research showing how probiotics and prebiotics enhance gut health and maximize nutrient absorption. These changes in intestinal morphology could lead to improved overall performance and digestive efficiency of poultry (Lambo et al., 2021; Latha et al., 2016; Lindgren and Dobrogosz, 1990). While shorter villi and deeper crypts can negatively affect nutrient absorption and performance, greater numbers of villi can improve nutrient absorption by increasing the intestinal surface area (Lipiński et al., 2021). Further investigations revealed that *Lactobacillus spp*. increased with probiotics supplementation, but there was no significant variation in *E. coli* counts. According to Dittoe et al. (2018) and Wang et al. (2015), probiotics such as *Lactobacillus* generate lactic acid, which makes the environment less conducive for pathogenic bacteria such as *Salmonella* spp.*, L. monocytogenes,* and *E. coli*. According to Bermudez-Brito et al. (2012), probiotics can prevent pathogen attachment to intestinal epithelial binding sites via competitive inhibition. Additionally, Callaway et al. (2008) reported that probiotics can decrease the growth and population of infections. The probiotics likely augmented the gut microbiota to promote the proliferation of advantageous microorganisms, hence improving gut health (Gernat et al., 2021; Manie et al., 1998).

Improvements in nutrient absorption may be linked to changes in gut architecture and function. In this study, the improvements in crude protein digestibility and nitrogen retention with pre- and probiotics supplementation may be related to the impact of dietary treatments on metabolic efficiency and gut microbial balance. These findings are consistent with earlier studies showing that nutritional supplements can maximize digestion and nutrient use (Afsharmanesh and Sadaghi, 2014) (Blajman et al., 2015). The observed improvement may be ascribed to the beneficial impact of probiotics on the balance of the gut microbiota and the reduction in deleterious metabolites that limit nutrients metabolism (Ribeiro et al., 2014). It was equally reported that probiotics enhance nitrogen retention, which may lead to a better ability to use dietary protein for maintenance and growth (Zhao et al., 2017; Zoghi et al., 2014). Additionally, synbiotics have also been demonstrated to increase nutrient absorption by enhancing gut architecture and functionality (Bai et al., 2013). Furthermore, supplementing with synbiotics was observed to increase the general activity level of turkeys. A tendency for increased feeding frequency was also noted in the behavioural patterns of turkeys supplemented with probiotics, prebiotics and synbiotics, suggesting that these nutritional interventions may impact behavior and appetite. These results are consistent with previous studies that have found that dietary changes may help optimize feeding schedules and energy levels in poultry (Bermudez-Brito et al., 2012), such that feeding with prebiotics may increase hunger and feeding behaviour (Elshaghabee et al., 2017; Wells, 2011).

## 5 Conclusion

This study demonstrates that synbiotic supplementation is a superior strategy for enhancing turkey productivity compared to probiotics or prebiotics alone. The synbiotic group exhibited the most significant improvements in overall growth performance and protein digestibility, underscoring the synergistic benefits of combining these supplements. While individual probiotics and prebiotics also showed positive effects such as modulating the lipid profile, improving nitrogen retention, and increasing beneficial cecal *Lactobacillus* populations—the effects were most pronounced in the synbiotic treatment. Overall, these findings provide strong evidence that synbiotics are an effective dietary intervention for optimizing nutrient utilization and growth efficiency in turkey production, offering a practical approach to improving poultry health and productivity.

## 6 Limitations of the study

While this study provides valuable insights into the effects of synbiotics, several limitations should be considered. First, the number of replicates, while consistent with some studies in the field, may limit the statistical power to detect more subtle treatment effects. Future research with a higher number of replicates would be beneficial to confirm these findings. Second, our assessment of immune status was based on organ weights and hematological parameters; functional immune assays, such as specific antibody titers or cytokine profiling, would provide a deeper understanding of the immune-modulatory effects. Finally, the experiment was conducted under specific management and environmental conditions; further validation in different commercial settings would help confirm the broad applicability of the results.

## Data Availability

The raw data supporting the conclusions of this article will be made available by the authors, without undue reservation.

## References

[B1] Abdelqader A. Abuajamieh M. Hayajneh F. Al-Fataftah A. R. (2020). Probiotic bacteria maintain normal growth mechanisms of heat stressed broiler chickens. J. Therm. Biol. 92, 102654. 10.1016/j.jtherbio.2020.102654 32888580

[B2] Afsharmanesh M. Sadaghi B. (2014). Effects of dietary alternatives (probiotic, green tea powder, and Kombucha tea) as antimicrobial growth promoters on growth, ileal nutrient digestibility, blood parameters, and immune response of broiler chickens. Comp. Clin. Pathol. 23, 717–724. 10.1007/s00580-013-1676-x

[B3] AOAC International (2025). Official methods of analysis. (Rockville, MD: AOAC International. Available online at: https://www.aoac.org/.

[B4] Asgari F. Madjd Z. Falak R. Bahar M. A. Nasrabadi M. H. Raiani M. (2016). Probiotic feeding affects T cell populations in blood and lymphoid organs in chickens. BM 7 (5), 669–675. 10.3920/BM2016.0014 27349931

[B5] Ashraf R. Shah N. P. (2014). Immune system stimulation by probiotic microorganisms. Crit. Rev. food Sci. Nutr. 54 (7), 938–956. 10.1080/10408398.2011.619671 24499072

[B6] Awad E. Awaad A. (2017). Role of medicinal plants on growth performance and immune status in fish. Fish and shellfish Immunol. 67, 40–54. 10.1016/j.fsi.2017.05.034 28526570

[B7] Bai S. Wu A. Ding X. Lei Y. Bai J. Zhang K. (2013). Effects of probiotic-supplemented diets on growth performance and intestinal immune characteristics of broiler chickens. Poult. Sci. 92 (3), 663–670. 10.3382/ps.2012-02813 23436517

[B8] Bermudez-Brito M. Plaza-Díaz J. Muñoz-Quezada S. Gómez-Llorente C. Gil A. (2012). Probiotic mechanisms of action. Ann. Nutr. Metabolism 61 (2), 160–174. 10.1159/000342079 23037511

[B9] Blajman J. Gaziano C. Zbrun M. V. Soto L. Astesana D. Berisvil A. (2015). *In vitro* and *in vivo* screening of native lactic acid bacteria toward their selection as a probiotic in broiler chickens. Res. Veterinary Sci. 101, 50–56. 10.1016/j.rvsc.2015.05.017 26267089

[B10] Callaway T. Edrington T. Anderson R. Harvey R. Genovese K. Kennedy C. (2008). Probiotics, prebiotics and competitive exclusion for prophylaxis against bacterial disease. Animal health Res. Rev. 9 (2), 217–225. 10.1017/S1466252308001540 19102792

[B11] Carr A. Maggini S. (2017). Vitamin C and immune function. Nutrients 9 (11), 1211. 10.3390/nu9111211 29099763 PMC5707683

[B12] Cheng X. Ning Z. (2023). Research progress on bird eggshell quality defects: a review. Poult. Sci. 102 (1), 102283. 10.1016/j.psj.2022.102283 36399932 PMC9673113

[B13] Cloft S. E. Robison C. I. Karcher D. M. (2018). Calcium and phosphorus loss from laying hen bones autoclaved for tissue removal. Poult. Sci. 97 (9), 3295–3297. 10.3382/ps/pey201 29800483

[B14] Dittoe D. K. Ricke S. C. Kiess A. S. (2018). Organic acids and potential for modifying the avian gastrointestinal tract and reducing pathogens and disease. Front. veterinary Sci. 5, 216. 10.3389/fvets.2018.00216 30238011 PMC6136276

[B15] Dittoe D. K. Atchley J. A. Feye K. M. Lee J. A. Knueven C. J. Ricke S. C. (2019). The efficacy of sodium Bisulfate salt (SBS) alone and combined with peracetic acid (PAA) as an antimicrobial on whole chicken drumsticks artificially inoculated with salmonella enteritidis. Front. Vet. Sci. 6, 6. 10.3389/fvets.2019.00006 30761312 PMC6363672

[B16] Elshaghabee F. M. F. Rokana N. Gulhane R. D. Sharma C. Panwar H. (2017). Bacillus as potential probiotics: status, concerns, and future perspectives. Front. Microbiol. 8, 1490. 10.3389/fmicb.2017.01490 28848511 PMC5554123

[B17] Fairbrother J. M. Nadeau É. Gyles C. L. (2005). *Escherichia coli* in postweaning diarrhea in pigs: an update on bacterial types, pathogenesis, and prevention strategies. Animal health Res. Rev. 6 (1), 17–39. 10.1079/ahr2005105 16164007

[B18] Feye K. M. Anderson K. L. Scott M. F. McIntyre D. R. Carlson S. A. (2016). Inhibition of the virulence, antibiotic resistance, and fecal shedding of multiple antibiotic-resistant Salmonella typhimurium in broilers fed original XPC^TM^ . Poult. Sci. 95 (12), 2902–2910. 10.3382/ps/pew254 27566726 PMC5144663

[B19] Gernat A. A. Santos F. B. O. Grimes J. L. (2021). Alternative approaches to antimicrobial use in the Turkey industry: challenges and perspectives. Ger. J. Vet. Res. 1 (3), 37–47. 10.51585/gjvr.2021.3.0018

[B20] Grundy S. M. Stone N. J. Bailey A. L. Beam C. Birtcher K. K. Blumenthal R. S. (2019). 2018 AHA/ACC/AACVPR/AAPA/ABC/ACPM/ADA/AGS/APhA/ASPC/NLA/PCNA guideline on the management of blood cholesterol: a report of the American college of cardiology/american heart association task Force on Clinical Practice fuidelines. J. Am. Coll. Cardiol. 73 (24), e285–e350. 10.1016/j.jacc.2018.11.003 30423393

[B21] Gulmez N. Bingol S. Deprem T. Koral Tasci S. Gulmez M. (2019). The Effect of Dietary Inclusion of Probiotics on Growth and Intestinal Morphology of Broiler Chickens. jwpr 9 (1), 24–31. 10.36380/jwpr.2019.3

[B22] Hikvision Digital Technology Co., Ltd. (2023). DS-2CD2146G1-IS [Surveillance camera]. Hangzhou, Zhejiang, China: Hikvision Digital Technology Co., Ltd.

[B23] Idowu P. A. Mpofu T. J. Magoro A. M. Modiba M. C. Nephawe K. A. Mtileni B. (2025). Impact of probiotics on chicken gut microbiota, immunity, behavior, and productive performance—a systematic review. Front. Anim. Sci. 6, 1562527. 10.3389/fanim.2025.1562527

[B24] Institute of Poultry Diseases, Faculty of Veterinary Medicine (2021). Turkey production and health: current challenges. Ger. J. Vet. Res. 1 (1), 3–14. 10.51585/gjvr.2021.0002

[B25] Joyce S. A. Shanahan F. Hill C. Gahan C. G. (2014). Bacterial bile salt hydrolase in host metabolism: Potential for influencing gastrointestinal microbe-host crosstalk. Gut Microbes 5 (5), 669–674. 10.4161/19490976.2014.969986 25483337 PMC4615832

[B26] Khomayezi R. Adewole D. (2022). Probiotics, prebiotics, and synbiotics: an overview of their delivery routes and effects on growth and health of broiler chickens. World’s Poult. Sci. J. 78 (1), 57–81. 10.1080/00439339.2022.1988804

[B27] Kunvar S. Czarnomska S. Pertoldi C. Tokarska M. (2021). In Search of Species-Specific SNPs in a Non-Model Animal (European Bison (Bison bonasus))—Comparison of De Novo and Reference-Based Integrated Pipeline of STACKS Using Genotyping-by-Sequencing (GBS) Data. Animals 11 (8), 2226. 10.3390/ani11082226 34438684 PMC8388393

[B28] Lambo M. T. Chang X. Liu D. (2021). The recent trend in the use of multistrain probiotics in livestock production: An overview. Animals 11 (10), 2805. 10.3390/ani11102805 34679827 PMC8532664

[B29] Latha S. Vinothini G. John Dickson Calvin D. Dhanasekaran D. (2016). *In vitro* probiotic profile based selection of indigenous actinobacterial probiont Streptomyces sp. JD9 for enhanced broiler production. J. Biosci. Bioeng. 121 (1), 124–131. 10.1016/j.jbiosc.2015.04.019 26111601

[B30] Li H. Zhang L. Chen L. Zhu Q. Wang W. Qiao J. (2016). Lactobacillus acidophilus alleviates the inflammatory response to enterotoxigenic *Escherichia coli* K88 via inhibition of the NF-κB and p38 mitogen-activated protein kinase signaling pathways in piglets. BMC Microbiol. 16 (1), 273. 10.1186/s12866-016-0862-9 27832756 PMC5105324

[B31] Lindgren S. E. Dobrogosz W. J. (1990). Antagonistic activities of lactic acid bacteria in food and feed fermentations. FEMS Microbiol. Lett. 87 (1–2), 149–163. 10.1111/j.1574-6968.1990.tb04885.x 2125429

[B32] Lipiński K. Mazur-Kuśnirek M. Antoszkiewicz Z. Makowski Z. Śliżewska K. Siwicki A. (2021). The effect of synbiotics and probiotics on the growth performance, gastrointestinal function and health status of turkeys. Archives Animal Nutr. 75 (5), 376–388. 10.1080/1745039X.2021.1958646 34459292

[B33] Lomax A. R. Calder P. C. (2008). Prebiotics, immune function, infection and inflammation: a review of the evidence. Br. J. Nutr. 101 (5), 633–658. 10.1017/S0007114508055608 18814803

[B34] Manie T. Khan S. Brözel V. S. Veith W. J. Gouws P. A. (1998). Antimicrobial resistance of bacteria isolated from slaughtered and retail chickens in South Africa. Lett. Appl. Microbiol. 26 (4), 253–258. 10.1046/j.1472-765x.1998.00312.x 9633089

[B35] Markowiak P. Śliżewska K. (2017). Effects of Probiotics, Prebiotics, and Synbiotics on Human Health. Nutrients 9 (9), 1021. 10.3390/nu9091021 28914794 PMC5622781

[B36] Markowiak P. Śliżewska K. (2018). The role of probiotics, prebiotics and synbiotics in animal nutrition. Gut Pathog. 10 (1), 21. 10.1186/s13099-018-0250-0 29930711 PMC5989473

[B37] Mazzoni M. Petracci M. Meluzzi A. Cavani C. Clavenzani P. Sirri F. (2015). Relationship between pectoralis major muscle histology and quality traits of chicken meat. Poult. Sci. 94 (1), 123–130. 10.3382/ps/peu043 25577799

[B38] Minitab L. L. C. (2023). Minitab Statistical Software (Version 21.1). Available online at: https://www.minitab.com.

[B39] Mohammadigheisar M. Shirley R. B. Barton J. Welsher A. Thiery P. Kiarie E. (2019). Growth performance and gastrointestinal responses in heavy Tom turkeys fed antibiotic free corn-soybean meal diets supplemented with multiple doses of a single strain Bacillus subtilis probiotic (DSM29784)1. Poult. Sci. 98 (11), 5541–5550. 10.3382/ps/pez305 31180117

[B40] Mookiah S. Sieo C. C. Ramasamy K. Abdullah N. Ho Y. W. (2014). Effects of dietary prebiotics, probiotic and synbiotics on performance, caecal bacterial populations and caecal fermentation concentrations of broiler chickens. J. Sci. Food Agric. 94 (2), 341–348. 10.1002/jsfa.6365 24037967

[B41] National Research Council (1994). Nutrient requirements of poultry. 9th revised edition. Washington, DC: National Academies Press.

[B42] OECD, Food and Agriculture Organization of the United Nations (2024). OECD-FAO Agricultural Outlook 2024-2033. Paris, France: OECD. Available online at: https://www.oecd.org/en/publications/oecd-fao-agricultural-outlook-2024-2033_4c5d2cfb-en.html.

[B43] Ooi L. G. Liong M. T. (2010). Cholesterol-Lowering Effects of Probiotics and Prebiotics: A Review of *in Vivo* and *in Vitro* Findings. IJMS 11 (6), 2499–2522. 10.3390/ijms11062499 20640165 PMC2904929

[B44] Patterson J. Burkholder K. (2003). Application of prebiotics and probiotics in poultry production. Poult. Sci. 82 (4), 627–631. 10.1093/ps/82.4.627 12710484

[B45] Reuben R. C. Sarkar S. L. Roy P. C. Anwar A. Hossain M. A. Jahid I. K. (2021). Prebiotics, probiotics and postbiotics for sustainable poultry production. World’s Poult. Sci. J. 77 (4), 825–882. 10.1080/00439339.2021.1960234

[B46] Ribeiro Jr V. Albino L. Rostagno H. Barreto S. Hannas M. Harrington D. (2014). Effects of the dietary supplementation of Bacillus subtilis levels on performance, egg quality and excreta moisture of layers. Animal Feed Sci. Technol. 195, 142–146. 10.1016/j.anifeedsci.2014.06.001

[B61] Roberfroid M. (2018). Prebiotics: concept, definition, criteria, methodologies, and products, in Handbook of prebiotics and prebiotics ingredients: health benefits and food applications. Editors Gibson G. R. Roberfroid M. B. CRC Press), 1–22.

[B47] Roche Diagnostics R. (2006). Cobas c 111 [Blood analyzer]. Indianapolis, USA: Roche Diagnostics Corporation.

[B48] Rodak B. F. Fritsma G. A. Doig K. (2020). Hematology: Clinical Principles and Applications. 5th ed. Elsevier.

[B49] Schley P. D. Field C. J. (2002). The immune-enhancing effects of dietary fibres and prebiotics. Br. J. Nutr. 87 (S2), S221–S230. 10.1079/BJNBJN/2002541 12088522

[B50] Schneider C. A. Rasband W. S. Eliceiri K. W. (2012). NIH Image to ImageJ: 25 years of image analysis. Nat. Methods 9 (7), 671–675. 10.1038/nmeth.2089 22930834 PMC5554542

[B51] Sharma N. K. Choct M. Toghyani M. Laurenson YCSM Girish C. K. Swick R. A. (2018). Dietary energy, digestible lysine, and available phosphorus levels affect growth performance, carcass traits, and amino acid digestibility of broilers. Poult. Sci. 97 (4), 1189–1198. 10.3382/ps/pex405 29340638

[B52] Shehata A. A. Hafez H. M. (2024). “General Overview on Turkey Production,” in Turkey Diseases and Disorders Volume 1: Bacterial and Fungal Infectious Diseases. Editors Hafez H. M. Shehata A. A. (Cham: Springer International Publishing), 1–26.

[B53] Teirlynck E. Bjerrum L. Eeckhaut V. Huygebaert G. Pasmans F. Haesebrouck F. (2009). The cereal type in feed influences gut wall morphology and intestinal immune cell infiltration in broiler chickens. Br. J. Nutr. 102 (10), 1453–1461. 10.1017/S0007114509990407 19664304

[B62] Toghiani S. VanRaden P. M. VandeHaar M. J. Baldwin R. L. Weigel K. A. White H. M. (2014). Dry matter intake in US Holstein cows: exploring the genomic and phenotypic impact of milk components and body weight composite. J. Diary Sci. 107 (9), 7009–7021. 10.3168/jds.2023-24296 38754817

[B54] Underwood W. Anthony R. (2020). AVMA guidelines for the euthanasia of animals. 30. 2020–2021.

[B55] Vahabi-Asil O. Bouyeh M. Qotbi A. Kadim I. T. Seidavi A. Centoducati G. (2017). Effects of a prebiotic on growth performance, blood parameters and immunity response of turkeys fed low protein diets. EuropPoultSci 81, 1–12. 10.1399/eps.2017.196

[B56] Wang C. Chang T. Yang H. Cui M. (2015). Antibacterial mechanism of lactic acid on physiological and morphological properties of Salmonella Enteritidis, *Escherichia coli* and Listeria monocytogenes. Food control. 47, 231–236. 10.1016/j.foodcont.2014.06.034

[B57] Wells J. M. (2011). Immunomodulatory mechanisms of lactobacilli. Microb. Cell Fact. 10 (Suppl. 1), S17. 10.1186/1475-2859-10-S1-S17 21995674 PMC3231924

[B58] Zhao X. Zhen Z. Wang X. Guo N. (2017). Synergy of a combination of nisin and citric acid against *Staphylococcus aureus* and *Listeria monocytogenes* . Food Addit. and Contam. Part A 34 (12), 2058–2068. 10.1080/19440049.2017.1366076 28795907

[B59] Zheng A. Luo J. Meng K. Li J. Bryden W. L. Chang W. (2016). Probiotic (Enterococcus faecium) induced responses of the hepatic proteome improves metabolic efficiency of broiler chickens (Gallus gallus). BMC Genomics 17 (1), 89. 10.1186/s12864-016-2371-5 26830196 PMC4736614

[B60] Zoghi A. Khosravi-Darani K. Sohrabvandi S. (2014). Surface Binding of Toxins and Heavy Metals by Probiotics. MRMC 14 (1), 84–98. 10.2174/1389557513666131211105554 24329992

